# Development of a prediction model for poor outcomes after thrombolysis in mild non-disabling ischemic stroke

**DOI:** 10.3389/fneur.2026.1754895

**Published:** 2026-01-23

**Authors:** Xiaopan Cao, Zhijian Fu, Li Li, Li Ren, Yang Jiang, Xue Cong, Bing Xu, Xin Zhang

**Affiliations:** 1 Department of Neurology VIII, Shenyang First People’s Hospital, Shenyang, Liaoning, China; 2 Department of Neurology I, Shenyang First People’s Hospital, Shenyang, Liaoning, China; 3 Neurology Outpatient Department, Shenyang First People’s Hospital, Shenyang, Liaoning, China; 4 Department of Critical Care Medicine, Shenyang First People’s Hospital, Shenyang, Liaoning, China; 5 Department of Neurology, Shenyang Tenth People’s Hospital, Shenyang, Liaoning, China; 6 Department of Neurorehabilitation, Shenyang First People’s Hospital, Shenyang, Liaoning, China

**Keywords:** logistic models, modified Rankin Scale, nomograms, prognosis, ROC curve, stroke, ischemic, thrombolytic therapy

## Abstract

**Background:**

Mild non-disabling ischemic stroke (MNDIS) is increasingly treated with intravenous thrombolysis, yet a substantial proportion of patients still experience poor functional outcomes, and robust tools for individualized risk prediction are lacking.

**Methods:**

In this retrospective cohort study, we analyzed 713 consecutive MNDIS patients who received intravenous thrombolysis within 4.5 h of symptom onset at an advanced stroke center between January 1, 2022 and December 31, 2024. Poor outcome was defined as a 90-day modified Rankin Scale (mRS) score ≥2. Candidate predictors, including demographic, clinical, laboratory, hemodynamic and imaging variables, were first screened by univariable analysis and then entered into a stepwise multivariable logistic regression model (entry *p* < 0.05, removal *p* > 0.10). A nomogram incorporating independent predictors was constructed in R, and its performance was evaluated using receiver operating characteristic (ROC) analysis, bootstrap calibration, and decision curve analysis.

**Results:**

Of the 713 patients, 91 (12.8%) had poor 90-day outcomes (mRS 2–6) and 622 (87.2%) had good outcomes (mRS 0–1). Admission NIHSS score (OR 1.37; 95% CI 1.10–1.72), 24-h NIHSS score (OR 1.78; 95% CI 1.52–2.10), diastolic blood pressure (OR 1.02 per mmHg; 95% CI 1.00–1.05), and coronary heart disease (OR 1.88; 95% CI 1.05–3.35) were independently associated with poor outcome. The resulting nomogram showed good discrimination (AUC 0.835; 95% CI 0.805–0.861; sensitivity 71.4%; specificity 84.1%), excellent calibration (bootstrap mean absolute error 0.014), and provided positive net clinical benefit across a wide range of risk thresholds (0.03–0.89).

**Conclusion:**

Admission and 24-h NIHSS scores, diastolic blood pressure, and coronary heart disease are key predictors of poor 90-day outcomes after thrombolysis in patients with MNDIS. The derived nomogram offers accurate, well-calibrated, and clinically useful individualized risk estimation, and may assist clinicians in early post-thrombolysis risk stratification and tailoring the intensity of monitoring and follow-up.

## 1 Introduction

Stroke is the second leading cause of death worldwide after ischemic heart disease (1). In 2019, the crude stroke mortality rate in China was 153.9 per 100,000. Acute ischemic stroke (AIS) is the most common neurological disorder in clinical practice, accounting for approximately 80% of all strokes and characterized by high rates of disability and death. The 1995 NINDS t-PA trial and the ECASS III trial demonstrated that timely intravenous thrombolysis within a 4.5-h window significantly improves outcomes in AIS, and the U. S. FDA approved intravenous recombinant tissue plasminogen activator (rt-PA) for AIS within 4.5 h of symptom onset. Mild ischemic stroke (MIS) is common in AIS: approximately half of AIS cases in the United States and about one third in China are MIS (2–5).

Traditionally, patients with MIS were often excluded from intravenous thrombolysis because symptoms were mild, the clinical course was perceived as benign, and clinicians were concerned about hemorrhagic complications (6). However, observational data indicate that approximately one third of patients with MIS are not fully independent at 90 days and remain functionally disabled. Consequently, thrombolysis rates in MIS have increased, and MIS is no longer considered an absolute contraindication to intravenous rt-PA. Not all patients benefit, however; a proportion remain disabled and some die despite treatment. Therefore, many clinical prediction models have been developed to estimate functional outcomes after thrombolysis, with the goal of supporting early prognostication and post-treatment management (7, 8).

In recent years, clinical prediction models for post-thrombolysis outcomes have proliferated, with nomograms becoming a research focus and widely disseminated online and in medical journals. A nomogram is a graphical statistical tool—essentially a visualization of a complex mathematical formula—typically derived from traditional methods such as multivariable logistic regression or Cox proportional hazards analysis. By integrating multiple prognostic and determinant variables, it yields a continuous probability of a specific event and provides individualized estimates based on a patient’s characteristics rather than group averages (9, 10). As a component of modern clinical decision-making, nomograms enable rapid, accurate, and intuitive risk quantification via digital interfaces, offering decision support and advancing personalized care. To date, they have been widely applied in stroke, oncology, surgery, and other specialties (11, 12). However, factors associated with poor outcomes after thrombolysis in mild non-disabling ischemic stroke (MNDIS) remain unclear, and no accurate, reliable nomogram has been established for this population. Accordingly, we enrolled thrombolysed MNDIS patients to identify risk factors for poor outcomes and to develop a simple, reliable nomogram; we then evaluated its predictive performance to enable early, individualized prediction of 90-day poor outcomes after intravenous thrombolysis in MNDIS.

## 2 Materials and methods

### 2.1 Study population

All patients were treated at the Advanced Stroke Center of Shenyang First People’s Hospital. We retrospectively analyzed clinical data from 713 consecutive patients with mild non-disabling ischemic stroke (MNDIS) who received intravenous thrombolysis at our center between January 1, 2022, and December 31, 2024. Diagnostic, inclusion, exclusion, and withdrawal criteria followed current national and international AIS guidelines and institutional protocols. Patients with a pre-stroke mRS score >1 were excluded to minimize confounding from pre-existing disability when defining 90-day functional outcomes. Pre-stroke mRS was determined at admission based on information from the patient and/or caregivers and corroborated with the electronic medical record. Intravenous thrombolytic dosing was categorized as low dose (0.6 mg/kg, maximum total dose 60 mg) and standard dose (0.9 mg/kg, maximum total dose 90 mg). Treatment decisions were made by the emergency physician in accordance with current stroke guidelines and in consultation with the on-call stroke neurologist. This study was reviewed and approved by the Ethics Committee of our medical center and conducted in accordance with the Declaration of Helsinki and relevant Chinese regulations and guidelines. Written informed consent was obtained from all patients or their legally authorized representatives.

Definition of MNDIS: Mild non-disabling ischemic stroke (MNDIS) was defined as acute ischemic stroke with a baseline NIHSS score ≤5 and the absence of a disabling neurological deficit at presentation. A disabling deficit was predefined as a deficit that, if unchanged, would prevent the patient from performing basic activities of daily living (ADL) or returning to work, consistent with commonly used definitions in thrombolysis studies of minor stroke. Operationally, patients were considered to have a disabling deficit if any of the following were present at baseline: complete hemianopia (NIHSS item 3 ≥ 2), severe aphasia (item 9 ≥ 2), neglect (item 11 ≥ 1), significant limb weakness (item 5 or 6 ≥ 2), or inability to walk independently (item 8 ≥ 2). The assessment of “non-disabling” status was performed by the treating stroke neurologist at presentation; in cases of uncertainty, two stroke neurologists reviewed the record and disagreements were resolved by consensus.

Treatment decision (IVT vs. non-IVT): In our center, all suspected AIS patients presenting within 4.5 h underwent standard emergency stroke evaluation (neurological examination with NIHSS, non-contrast CT to exclude intracranial hemorrhage, and assessment of contraindications to thrombolysis). For patients meeting the MNDIS definition and arriving within the time window, IVT was offered according to current guideline recommendations after a risk–benefit discussion. The final decision to proceed with IVT was made jointly by the emergency physician and the on-call stroke neurologist, together with the patient and/or legally authorized representative after informed consent. Patients who did not receive IVT (e.g., contraindications, refusal after shared decision-making, or other protocol-based reasons) were treated with antiplatelet therapy per guideline-based secondary prevention (including dual antiplatelet therapy when clinically indicated).

### 2.2 Study methods

All MNDIS patients treated with IVT who were included in this study were followed up for 90 days. Patients lost to follow-up during this period were excluded. According to the modified Rankin Scale (mRS) score at 90 days, patients were divided into a poor prognosis group and a favorable prognosis group: an mRS score ≥2 was defined as poor prognosis, and an mRS score ≤1 was defined as favorable prognosis. For patients meeting the MNDIS inclusion criteria and with complete clinical data after IVT, univariate and multivariate analyses were performed. Multivariate logistic regression was used to identify independent risk factors for poor prognosis after thrombolysis in MNDIS patients, with a stepwise selection method applied. Based on the identified predictors of poor outcome, a nomogram model for predicting poor prognosis after thrombolysis in MNDIS patients was constructed using R software packages. The accuracy of the model and its clinical net benefit were evaluated by bootstrap resampling and clinical decision curve analysis.

### 2.3 Data collection

Basic demographic information: age, sex, and body weight. Past history related to the index cerebrovascular event: smoking, hypertension, hyperlipidemia, coronary artery disease, diabetes mellitus, atrial fibrillation, prior stroke, and transient ischemic attack (TIA). Pre-stroke medication use: antiplatelet agents and anticoagulants. Laboratory and hemodynamic parameters: mean blood glucose, systolic blood pressure, diastolic blood pressure, total cholesterol, high-density lipoprotein cholesterol, low-density lipoprotein cholesterol, and triglycerides. Hemorrhagic events after thrombolysis: hemorrhagic transformation, symptomatic intracranial hemorrhage, asymptomatic intracranial hemorrhage, absence of hemorrhage, and the subtype of hemorrhagic transformation (HI1, HI2, PH1, PH2).

Thrombolytic regimen and time parameters: thrombolytic dose (0.9 mg/kg or 0.6 mg/kg) and time from symptom onset to emergency treatment. Neurological severity and stroke etiology: NIHSS score at onset, NIHSS score at 24 h, and stroke etiology according to the TOAST classification (cardioembolism, small-artery occlusion, large-artery atherosclerosis, undetermined etiology, and other determined causes).

### 2.4 Study outcomes

For MNDIS patients treated with intravenous thrombolysis, 90-day outcomes were assessed using the modified Rankin Scale (mRS) to evaluate neurological recovery and mortality. Favorable outcome: mRS score 0–1 at 90 days after thrombolysis. Poor outcome: mRS score 2–6 at 90 days after thrombolysis. The mRS scoring criteria were as follows; 0: No symptoms at all; 1: No significant disability despite symptoms; able to carry out all usual duties and activities; 2: Slight disability; unable to carry out all previous activities but able to look after own affairs without assistance; 3: Moderate disability; requires some help but able to walk without assistance; 4: Moderately severe disability; unable to attend to own bodily needs without assistance and unable to walk unassisted; 5: Severe disability; bedridden, incontinent, and requiring constant nursing care and attention; 6: Death.

### 2.5 Statistical analysis

All statistical analyses were performed using Python (version 3.11) and R software (version 4.1.1). Continuous variables with a normal distribution are presented as mean ± standard deviation, whereas non-normally distributed data are summarized as median (interquartile range, IQR). Categorical variables are expressed as counts (percentages). A nomogram was constructed in R based on multivariable logistic regression. Multivariable logistic regression with stepwise selection was applied, with an entry criterion of 0.05 and a removal criterion of 0.10. Receiver operating characteristic (ROC) curves were plotted in Python to validate the nomogram, and the area under the curve (AUC), sensitivity, specificity, and Youden index were calculated. Model calibration was assessed using the bootstrap method, and calibration plots were generated in Python. Decision curve analysis was performed in Python to evaluate the clinical utility of the model. A two-sided *p* value < 0.05 was considered statistically significant. Results from logistic regression are reported as odds ratios (ORs) with 95% confidence intervals; ORs reflect changes in odds and should not be interpreted as risk ratios.

## 3 Results

### 3.1 Baseline characteristics

The patient screening and treatment allocation process is summarized in Supplementary Figure S1. Briefly, among 7,449 adult patients clinically diagnosed with ischemic stroke, 1,905 presented within 4.5 h from symptom onset. After applying prespecified exclusion criteria, 1,122 patients were registered, of whom 740 received IVT and 382 did not. After excluding patients with missing 90-day mRS or loss to follow-up, 713 IVT-treated patients were included in the final analysis. Specifically, patients with pre-stroke mRS > 1 were excluded (Supplementary Figure S1). A total of 713 MNDIS patients treated with intravenous thrombolysis were included in the statistical analysis. The median age was 63 years (IQR: 57–69), and 73.07% of the patients were male. Other baseline characteristics were as follows: smoking, 48.67%; hypertension, 62.41%; hyperlipidemia, 22.86%; coronary artery disease, 23.28%; diabetes mellitus, 28.47%; atrial fibrillation, 6.59%; prior stroke, 26.23%; prior transient ischemic attack (TIA), 1.96%; use of antiplatelet agents, 21.6%; and use of anticoagulants, 0.42%. According to the TOAST classification, the proportions of etiologic subtypes were: cardioembolism, 3.23%; small-artery occlusion, 28.33%; large-artery atherosclerosis, 61.01%; undetermined etiology, 7.01%; and other determined etiology, 0.42%. The overall rate of hemorrhagic transformation was 3.93%, including symptomatic intracranial hemorrhage in 1.68% and asymptomatic intracranial hemorrhage in 2.24%. With respect to hemorrhagic transformation subtypes, no hemorrhage occurred in 96.07%, HI1 in 3.23%, HI2 in 0.42%, PH1 in 0.14%, and PH2 in 0.14% of patients. The proportions of thrombolytic dose were 57.64% for 0.6 mg/kg and 42.36% for 0.9 mg/kg. The medians (IQR) of lipid and metabolic parameters were as follows: high-density lipoprotein cholesterol, 0.86–1.21; low-density lipoprotein cholesterol, 2.60–3.69; triglycerides, 1.15–2.24; and total cholesterol, 4.20–5.69. The median blood glucose level was 6.2 mmol/L (IQR: 5.2–8.1). The median systolic blood pressure was 149 mmHg (IQR: 138–160), and the median diastolic blood pressure was 85 mmHg (IQR: 80–95). The median NIHSS score on admission was 2 (IQR: 1–3), and the median NIHSS score at 24 h was 1 (IQR: 0–2). The median body weight was 70 kg (IQR: 62–78), and the median onset-to-emergency treatment time was 106 min (IQR: 65–156.75). Details are presented in Table 1.

**Table 1:** Baseline characteristics of the patients.

Variable	*n* (%)/Median (IQR)
Male, *n* (%)	521 (73.07)
Smoking, *n* (%)	347 (48.67)
Hypertension, *n* (%)	445 (62.41)
Hyperlipidemia, *n* (%)	163 (22.86)
Coronary artery disease, *n* (%)	166 (23.28)
Diabetes mellitus, *n* (%)	203 (28.47)
Atrial fibrillation, *n* (%)	47 (6.59)
Previous stroke, *n* (%)	187 (26.23)
Previous TIA, *n* (%)	14 (1.96)
Antiplatelet agents, *n* (%)	154 (21.6)
Anticoagulants, *n* (%)	3 (0.42)
TOAST subtype, *n* (%) – Cardioembolism	23 (3.23)
TOAST subtype, *n* (%) – Small-artery occlusion	202 (28.33)
TOAST subtype, *n* (%) – Large-artery atherosclerosis	435 (61.01)
TOAST subtype, *n* (%) – Undetermined etiology	50 (7.01)
TOAST subtype, *n* (%) – Other determined etiology	3 (0.42)
Hemorrhagic transformation, *n* (%)	28 (3.93)
Symptomatic intracranial hemorrhage, *n* (%)	12 (1.68)
Asymptomatic intracranial hemorrhage, *n* (%)	16 (2.24)
Type of hemorrhagic transformation – No hemorrhage, *n* (%)	685 (96.07)
Type of hemorrhagic transformation – HI1, *n* (%)	23 (3.23)
Type of hemorrhagic transformation – HI2, *n* (%)	3 (0.42)
Type of hemorrhagic transformation – PH1, *n* (%)	1 (0.14)
Type of hemorrhagic transformation – PH2, *n* (%)	1 (0.14)
Thrombolytic dose – 0.6 mg/kg, *n* (%)	411 (57.64)
Thrombolytic dose – 0.9 mg/kg, *n* (%)	302 (42.36)
Age, median (IQR)	63 (57–69)
High-density lipoprotein cholesterol, median (IQR)	1.01 (0.86–1.21)
Low-density lipoprotein cholesterol, median (IQR)	3.14 (2.60–3.69)
Triglycerides, median (IQR)	1.54 (1.15–2.24)
Total cholesterol, median (IQR)	4.92 (4.20–5.69)
Blood glucose, median (IQR)	6.22 (5.20–8.10)
Systolic blood pressure, median (IQR)	149 (138–160)
Diastolic blood pressure, median (IQR)	85 (80–95)
NIHSS score on admission, median (IQR)	2 (1–3)
NIHSS score at 24 h, median (IQR)	1 (0–2)
Body weight, median (IQR)	70 (62–78)
Onset-to-emergency treatment time (min), median (IQR)	106 (65–156)

HI, hemorrhagic infarction; PH, parenchymal hematoma.

### 3.2 Comparison of baseline characteristics

MNDIS patients treated with IVT were divided into a poor prognosis group and a favorable prognosis group according to their outcomes, with 91 patients in the poor prognosis group and 622 patients in the favorable prognosis group. Baseline data were statistically analyzed and compared between the two groups. The results showed that the proportions of hypertension (*χ*^2^ = 4.55, *p* = 0.033) and diabetes mellitus (*χ*^2^ = 4.049, *p* = 0.044), as well as age (*Z* = −1.968, *p* = 0.049), NIHSS score on admission (*Z* = −6.051, *p* < 0.001), and NIHSS score at 24 h (*Z* = −10.131, *p* < 0.001), were all higher in the poor prognosis group than in the favorable prognosis group, with statistically significant differences. In contrast, the proportion of hyperlipidemia (*χ*^2^ = 4.35, *p* = 0.037) and the level of high-density lipoprotein cholesterol (*Z* = −1.965, *p* = 0.049) were lower in the poor prognosis group than in the favorable prognosis group, and these differences were also statistically significant. Details are shown in Table 2.

**Table 2:** Comparison of baseline characteristics between patients with favorable and poor outcomes.

Variable	Favorable outcome group (*n* = 622)	Poor outcome group (*n* = 91)	*Z*/*χ*^2^	*p* value
Male, *n* (%)	456 (73.31)	65 (71.43)	0.143	0.705
Smoking, *n* (%)	306 (49.20)	41 (45.05)	0.545	0.460
Hypertension, *n* (%)	379 (60.93)	66 (72.53)	4.55	0.033
Hyperlipidemia, *n* (%)	150 (24.12)	13 (14.29)	4.35	0.037
Coronary artery disease, *n* (%)	141 (22.67)	25 (27.47)	1.026	0.311
Diabetes mellitus, *n* (%)	169 (27.17)	34 (37.36)	4.049	0.044
Atrial fibrillation, *n* (%)	43 (6.91)	4 (4.40)	0.817	0.366
Previous stroke, *n* (%)	162 (26.05)	25 (27.47)	0.084	0.772
Previous TIA, *n* (%)	12 (1.93)	2 (2.20)	0.030	0.863
Antiplatelet agents, *n* (%)	132 (21.22)	22 (24.18)	0.409	0.522
Anticoagulants, *n* (%)	3 (0.48)	0 (0)	0.441	0.507
TOAST subtype, *n* (%)			6.509	0.164
Cardioembolism	19 (3.05)	4 (4.40)		
Small-artery occlusion	185 (29.74)	17 (18.68)		
Large-artery atherosclerosis	370 (59.49)	65 (71.43)		
Undetermined etiology	45 (7.23)	5 (5.49)		
Other determined etiology	3 (0.48)	0 (0)		
Hemorrhagic transformation, *n* (%)	22 (3.54)	6 (6.59)	1.966	0.161
Symptomatic intracranial hemorrhage, *n* (%)	10 (1.61)	2 (2.20)	0.167	0.683
Asymptomatic intracranial hemorrhage, *n* (%)	12 (1.93)	4 (4.40)	2.201	0.138
Type of hemorrhagic transformation, *n* (%)			4.476	0.345
No hemorrhage	600 (96.46)	85 (93.41)		
HI1	17 (2.73)	6 (6.59)		
HI2	3 (0.48)	0 (0)		
PH1	1 (0.16)	0 (0)		
PH2	1 (0.16)	0 (0)		
Thrombolytic dose			0.123	0.726
0.6 mg/kg, *n* (%)	357 (57.40)	54 (59.34)		
0.9 mg/kg, *n* (%)	265 (42.60)	37 (40.66)		
Age, years, median (IQR)	62 (57–69)	65 (58–73)	−1.968	0.049
High-density lipoprotein cholesterol, median (IQR)	1.01 (0.87–1.22)	0.95 (0.84–1.19)	−1.965	0.049
Low-density lipoprotein cholesterol, median (IQR)	3.15 (2.62–3.67)	3.08 (2.42–3.77)	−0.467	0.641
Triglycerides, median (IQR)	1.55 (1.15–2.26)	1.52 (1.16–2.13)	−0.329	0.742
Total cholesterol, median (IQR)	4.95 (4.24–5.71)	4.78 (3.92–5.65)	−1.431	0.152
Blood glucose, median (IQR)	6.23 (5.20–8.10)	6.20 (5.18–8.40)	−0.275	0.783
Systolic blood pressure, mmHg, median (IQR)	148 (138–160)	152 (140–167)	−1.932	0.053
Diastolic blood pressure, mmHg, median (IQR)	85 (80–94)	87 (80–100)	−1.353	0.176
NIHSS score on admission, median (IQR)	2 (1–3)	3 (2–4)	−6.051	<0.001
NIHSS score at 24 h, median (IQR)	1 (0–2)	4 (2–5)	−10.131	<0.001
Body weight, kg, median (IQR)	70 (62–78)	70 (61–77)	−0.146	0.884
Onset-to-emergency treatment time, min, median (IQR)	106 (62.5–155.5)	105 (72–161)	−0.740	0.460

### 3.3 Screening of prognostic factors

Multivariable logistic regression analysis was performed to identify factors associated with poor outcomes after thrombolysis in MNDIS patients. A stepwise selection method was used, with an entry criterion of 0.05 and a removal criterion of 0.10. The analysis showed that admission NIHSS score (*p* = 0.006), 24-h NIHSS score (*p* < 0.001), diastolic blood pressure (*p* = 0.026), and coronary artery disease (*p* = 0.033) were independent predictors of poor outcome.

Specifically, each 1-point increase in admission NIHSS score was associated with 37% higher odds of poor outcome (OR 1.37; 95% CI 1.10–1.72). Each 1-point increase in 24-h NIHSS score was associated with 78% higher odds of poor outcome (OR 1.78; 95% CI 1.52–2.10). Each 1-mmHg increase in diastolic blood pressure was associated with 2% higher odds of poor outcome (OR 1.02; 95% CI 1.00–1.05). Patients with coronary artery disease had higher odds of poor outcome compared with those without coronary artery disease (OR 1.88; 95% CI 1.05–3.35). Details are presented in Table 3.

**Table 3:** Independent risk factors for poor outcome.

Variable	Odds ratio (95% CI)	*p* value
Admission NIHSS score	1.37 (1.10–1.72)	0.006
24-h NIHSS score	1.78 (1.52–2.10)	<0.001
Diastolic blood pressure	1.02 (1.00–1.05)	0.026
Coronary heart disease	1.88 (1.05–3.35)	0.033

ORs for NIHSS are per 1-point increase; OR for diastolic blood pressure is per 1 mmHg; coronary heart disease is yes vs no.

### 3.4 Construction of the nomogram prediction model

**Figure 1:**
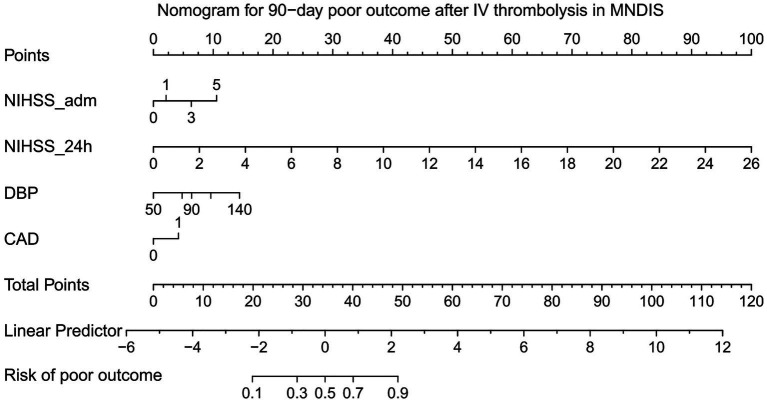
Nomogram for predicting poor prognosis.

Based on the independent risk factors for poor 90-day outcomes after thrombolysis in MNDIS patients identified by the aforementioned multivariable logistic regression analysis, a nomogram for predicting poor outcomes was constructed using the “rms” package in R. The resulting nomogram model is shown in Figure 1. In the nomogram, a vertical line is drawn from the value of each predictor on the horizontal axis to the “Points” scale to obtain the corresponding score. For an individual patient, the scores corresponding to the four variables—NIHSS score on admission, 24-h NIHSS score, diastolic blood pressure, and coronary heart disease—are summed to yield a total score, which represents that patient’s risk of poor outcome at 90 days.

### 3.5 ROC curve of the nomogram prediction model

**Figure 2:**
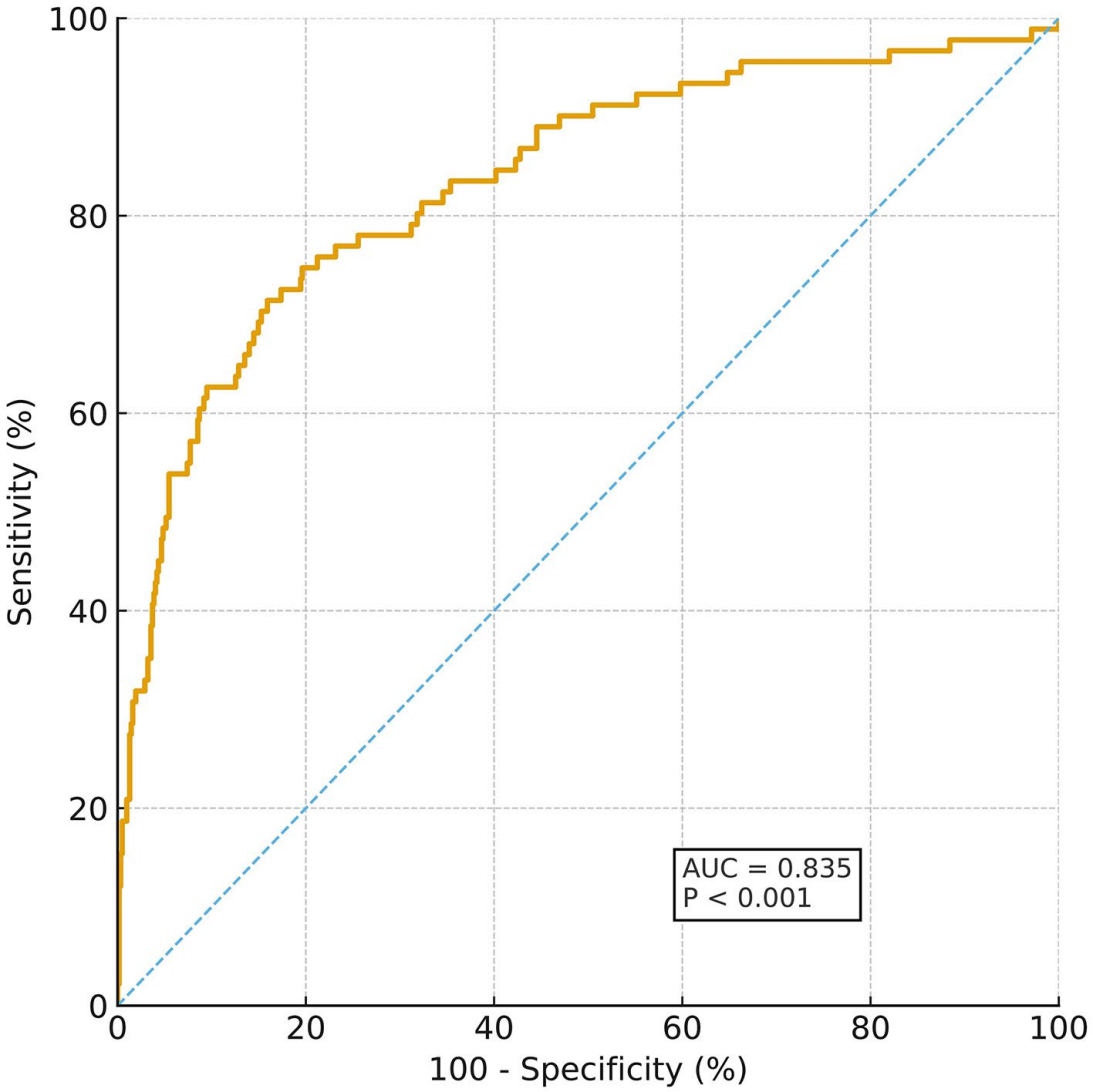
ROC curve for predicting poor outcome.

Taking sensitivity as the y-axis and 1 − specificity as the x-axis, the ROC curve of the nomogram prediction model was plotted. The ROC curve showed that the constructed nomogram had strong ability to predict poor 90-day outcomes after thrombolysis in MNDIS patients, with an area under the curve (AUC) of 0.835, 95% CI 0.805–0.861, *p* < 0.001, sensitivity 71.43%, specificity 84.08%, and a Youden index of 0.5551. The detailed results are shown in Figure 2.

### 3.6 Validation of the nomogram curve using the bootstrap method

**Figure 3:**
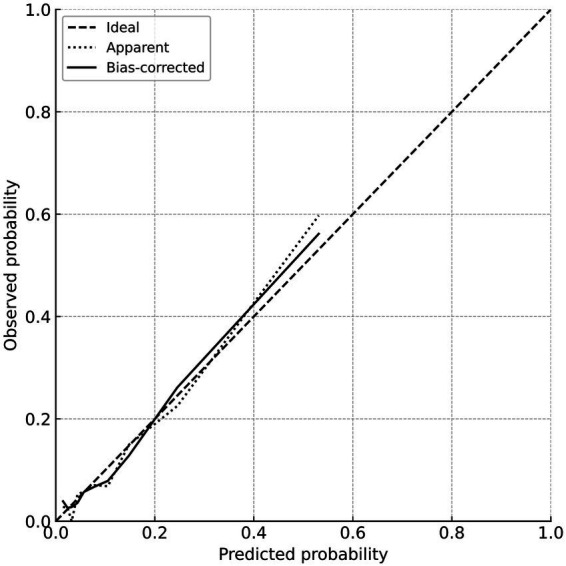
Calibration curve for predicting poor prognosis.

The calibration of the constructed clinical prediction model was evaluated using the bootstrap method. The calibration curve reflects the degree of agreement between the actual outcomes and the nomogram-predicted outcomes. The nomogram was validated by 1,000 bootstrap resamples, and the mean absolute error of the calibration curve was 0.014, indicating good agreement between the calibration curve and the ideal curve. The mean predicted probabilities in each group were essentially consistent with the observed values, demonstrating good calibration. The detailed results are shown in Figure 3.

### 3.7 Clinical decision curve of the nomogram model

**Figure 4:**
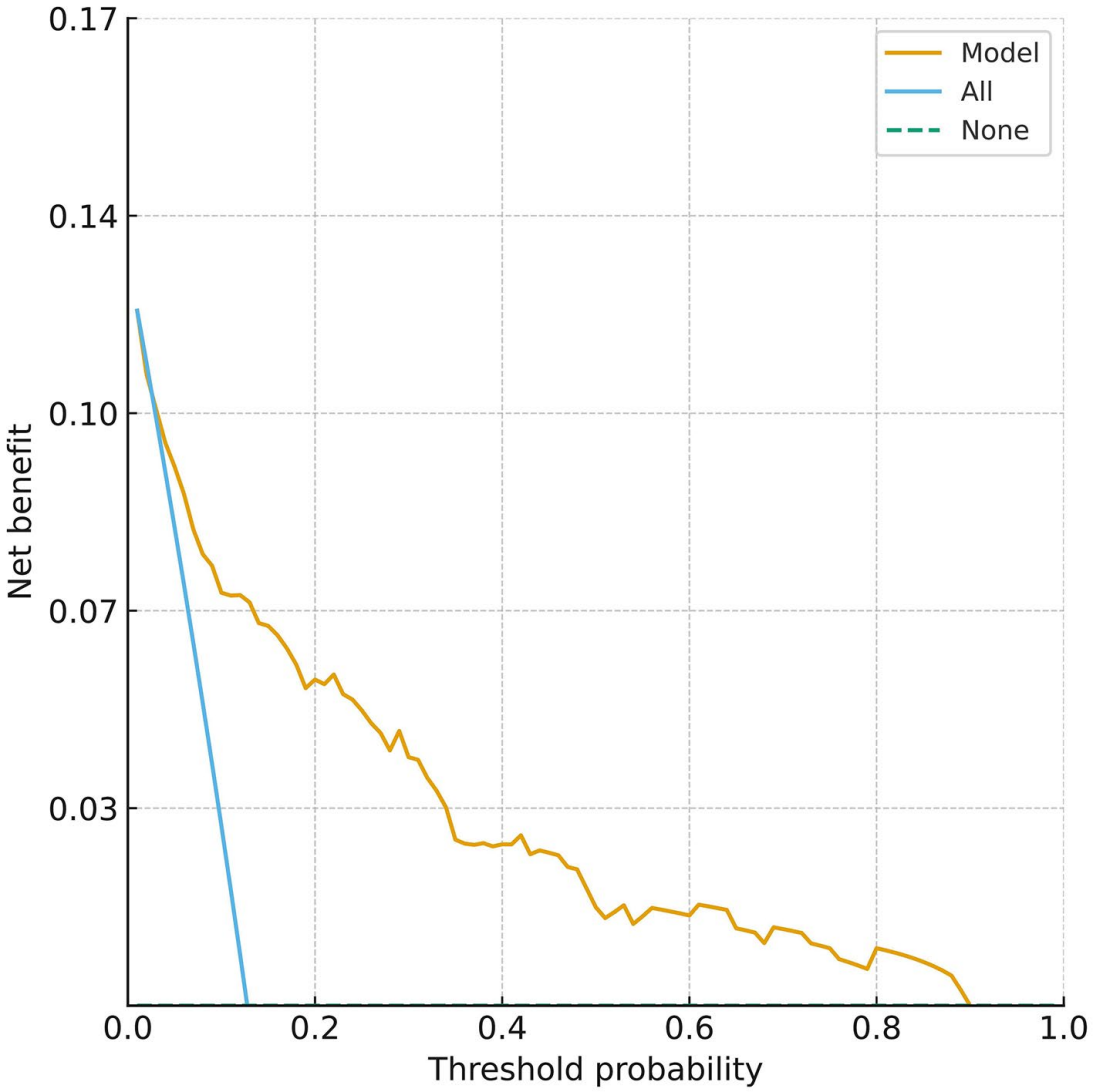
Decision curve for predicting poor prognosis.

The clinical decision curve of the nomogram model showed that using this nomogram to predict prognosis can provide a greater net clinical benefit. When the threshold probability for predicting poor 90-day outcome after thrombolysis in MNDIS patients is between 0.03 and 0.89, the applicability of this model is optimal. The specific results are shown in Figure 4.

## 4 Discussion

Stroke is the second leading cause of death worldwide after ischemic heart disease, and in 2019 the crude mortality rate from stroke in the Chinese population was 153.9 per 100,000 population (13). Acute ischemic stroke (AIS) is the predominant subtype of stroke and a major cause of severe disability and death. Intravenous thrombolysis is one of the most effective pharmacologic treatments for AIS; however, while it reduces disability, it also increases the risk of intracranial hemorrhage and neurological deterioration, and a proportion of patients ultimately experience poor functional outcomes or even death.

For the evaluation of outcomes after intravenous thrombolysis, the modified Rankin Scale (mRS) has become a widely used assessment tool in clinical studies, with a reported sensitivity and specificity of more than 80% (14, 15). In patients with mild ischemic stroke (MIS) receiving thrombolysis, baseline NIHSS scores are relatively low; therefore, several trials (such as PRISMS, ECASS, and ECASS II) have adopted mRS ≤ 1 to define a favorable functional outcome and mRS ≥ 2 to define an unfavorable outcome. In line with this evidence, the present study defined poor outcome as a 90-day mRS ≥ 2 and good outcome as a 90-day mRS ≤ 1, and on this basis constructed a nomogram to predict poor outcome after thrombolysis in patients with MNDIS, with the aim of enabling individualized risk prediction.

The NIH Stroke Scale (NIHSS) is currently the most widely used tool worldwide for assessing stroke severity. A multivariable analysis has shown that patients with early poor outcomes after intravenous thrombolysis tend to have higher NIHSS scores on admission, and NIHSS≥15 is negatively associated with early functional improvement (16). Multiple studies have confirmed that higher NIHSS scores at admission are associated with a lower likelihood of good outcome (17); in MIS cohorts, admission NIHSS has likewise been associated with unfavorable prognosis (18). Consistent with these findings, the present study identified both admission NIHSS score and 24-h NIHSS score as independent risk factors for poor outcome, in agreement with previous reports (19).

Blood pressure (BP) management is a key determinant of outcomes in the peri-thrombolytic period, yet the optimal strategy remains debated. In acute ischemic stroke, elevated BP may serve as a compensatory response to maintain cerebral perfusion and collateral flow, which underpins the concept of “permissive hypertension” in patients not undergoing reperfusion therapy (20). However, in patients treated with intravenous thrombolysis, persistently elevated BP and BP variability have been associated with worse functional outcomes and higher hemorrhagic risk in observational cohorts, supporting active BP control during and after thrombolysis. Current guidelines therefore recommend lowering BP to <185/110 mmHg before IV alteplase and maintaining <180/105 mmHg during and for the first 24 h after treatment, balancing safety (symptomatic intracranial hemorrhage) against potential hypoperfusion (21). Nevertheless, randomized evidence has yielded mixed results: the ENCHANTED BP arm showed that intensive SBP lowering (target 130–140 mmHg) was generally safe and reduced intracranial hemorrhage, but did not improve 90-day functional outcome compared with guideline-based control (<180 mmHg), highlighting the uncertainty around aggressive BP reduction (22). In this context, a pragmatic approach is to adhere to guideline upper limits, avoid hypotension, and minimize excessive BP fluctuations. Consistent with prior reports, our finding that higher diastolic BP was independently associated with 90-day poor outcome suggests that closer BP monitoring and avoidance of sustained BP elevation in the early post-thrombolysis period may be particularly important in MNDIS.

AIS and coronary heart disease (CHD) share a primary underlying pathology of systemic atherosclerosis, and CHD may therefore act as a composite marker of diffuse vascular burden and impaired vascular reserve. Prior work has shown that concomitant CHD is associated with worse stroke outcomes and a higher risk of subsequent major vascular events (23, 24). Beyond overt recurrent events, CHD may plausibly contribute to poorer functional recovery through several non-mutually exclusive mechanisms, including reduced cardiac output or subclinical myocardial ischemia leading to impaired cerebral perfusion/collateral support, greater susceptibility to hemodynamic instability during the acute and early recovery phases, and limitations in rehabilitation tolerance and cardiopulmonary reserve. In our cohort, classic vascular comorbidities (e.g., hypertension, diabetes mellitus, atrial fibrillation, smoking, prior stroke/TIA) were entered into the modeling process but were not retained in the final multivariable model, suggesting that their effects may be mediated by more proximate markers of acute neurological severity and early evolution (admission/24-h NIHSS and early blood pressure profile), collinearity among risk factors, and/or limited statistical power for less prevalent conditions (e.g., atrial fibrillation). Accordingly, the independent association of CHD in our model is unlikely to reflect only the occurrence of new vascular events; rather, it may also capture baseline systemic vascular disease severity and cardiac reserve that influence recovery even in the absence of clinically overt recurrent events.

Previous studies in broader AIS populations have suggested that advanced age, longer onset-to-treatment time, hyperglycemia/diabetes, atrial fibrillation, smoking, and a history of hypertension or prior stroke/TIA are associated with unfavorable outcomes after thrombolysis (25). In our thrombolysed MNDIS cohort, some of these comorbidities (notably hypertension and diabetes) showed associations with poor outcome in univariable comparisons; however, they were not retained in the final multivariable model. Several hypotheses may explain this discrepancy. First, in MNDIS, functional outcomes may be more strongly driven by proximate markers of acute neurological severity and early evolution (admission and 24-h NIHSS) and early hemodynamics, which can mediate or attenuate the apparent effects of baseline comorbidities after adjustment. Second, comorbidities cluster and may introduce collinearity (e.g., hypertension/diabetes with atherosclerotic burden), such that a single variable (e.g., CHD as a marker of systemic vascular disease and reduced reserve) may capture overlapping risk. Third, atrial fibrillation was relatively uncommon in this cohort and cardioembolic stroke accounted for a small proportion, which may have limited statistical power to detect an independent effect. Fourth, our comorbidity variables were largely binary and did not capture severity, duration, or control (e.g., HbA1c, long-term BP burden/variability, smoking pack-years), which may lead to nondifferential misclassification and bias effects toward the null. Finally, treatment selection and acute management may differ in MNDIS (including standardized thrombolysis protocols and secondary prevention), potentially diminishing the incremental prognostic contribution of baseline comorbidities. Future multicenter studies incorporating more granular comorbidity severity metrics and external validation are warranted.

The nomogram developed in this study showed good discrimination (AUC 0.835). It is important to clarify that previously published tools such as the Stroke–Thrombolytic Predictive Instrument (Stroke-TPI) by Kent et al. (26) and the START nomogram by Cappellari et al. (27) are primarily pre-treatment (baseline) prediction tools, built on variables available before thrombolysis to predict post-thrombolysis functional outcomes. In contrast, our model was intentionally developed as an early post-treatment prediction tool by incorporating the 24-h NIHSS, which captures early neurological evolution after IVT. Because these models operate at different time points and leverage different information sets, their discrimination metrics are not directly comparable, and improved performance is expected when post-treatment variables are included. Therefore, Stroke-TPI/START may be more suitable for pre-IVT counseling/selection, whereas our nomogram is intended for early post-thrombolysis risk stratification to tailor monitoring intensity and downstream management.

Clinical implications and intended use of the nomogram: We agree that the present nomogram is primarily a post-treatment tool because it incorporates the 24-h NIHSS, and therefore it is not intended to guide initial patient selection for IVT. Rather, its clinical value lies in early post-thrombolysis risk stratification to tailor the intensity of monitoring and downstream management. For example, if a patient is predicted to have a low likelihood of achieving mRS 0–1 at 90 days (i.e., a higher predicted risk of poor outcome), clinicians may consider: (1) closer neurological observation in a stroke unit, (2) earlier repeat neuroimaging or vascular evaluation when clinically indicated to identify early neurological deterioration or complications, (3) more stringent management of physiological parameters (e.g., blood pressure) and comorbid conditions such as coronary heart disease, and (4) earlier initiation of multidisciplinary rehabilitation planning and closer follow-up after discharge. Conversely, patients predicted to be at low risk may be suitable for standard monitoring and streamlined discharge planning. In this context, the decision curve analysis can be interpreted as the net benefit of using the nomogram to trigger enhanced post-thrombolysis management across clinically relevant risk thresholds.

Nonetheless, this study has several limitations. It was a single-center study with a relatively limited sample size; only Chinese patients were included, and external validation in other populations is lacking. In addition, the model was based mainly on clinical variables and has not yet integrated imaging markers or biomarkers. In addition, because the model incorporates the 24-h NIHSS, it is intended for post-thrombolysis prognostication rather than pre-treatment selection for IVT. Future work should focus on external validation and updating of the model in multicenter, large-scale, and multiethnic cohorts, and on exploring the integration of the nomogram into electronic medical records or mobile platforms to further enhance its usability in clinical practice. We did not have detailed metrics of comorbidity severity/control (e.g., HbA1c, long-term BP variability, smoking intensity), which may have limited the ability to detect independent associations for these factors. Finally, recurrent vascular events during follow-up were not systematically adjudicated; therefore, we could not formally disentangle whether the effect of CHD on 90-day functional outcome was mediated by recurrent events or by baseline cardiac/vascular reserve.

## 5 Conclusion

The risk factors for poor prognosis after thrombolysis in patients with MNDIS are baseline NIHSS score, 24-h NIHSS score, diastolic blood pressure, and coronary heart disease. The nomogram model constructed based on these factors exhibits good clinical predictive value; it can estimate the individual probability of poor outcome according to the contribution of each factor and may assist clinicians in the evaluation and management of these patients.

## Data Availability

The raw data supporting the conclusions of this article will be made available by the authors, without undue reservation.
