# Supplementary figures and images for: Development of a prediction model for poor outcomes after thrombolysis in mild non-disabling ischemic stroke
Source: Front Neurol. 2026 Jan 23;17:1754895. doi: 10.3389/fneur.2026.1754895 (PMC12875937; doi:10.3389/fneur.2026.1754895)

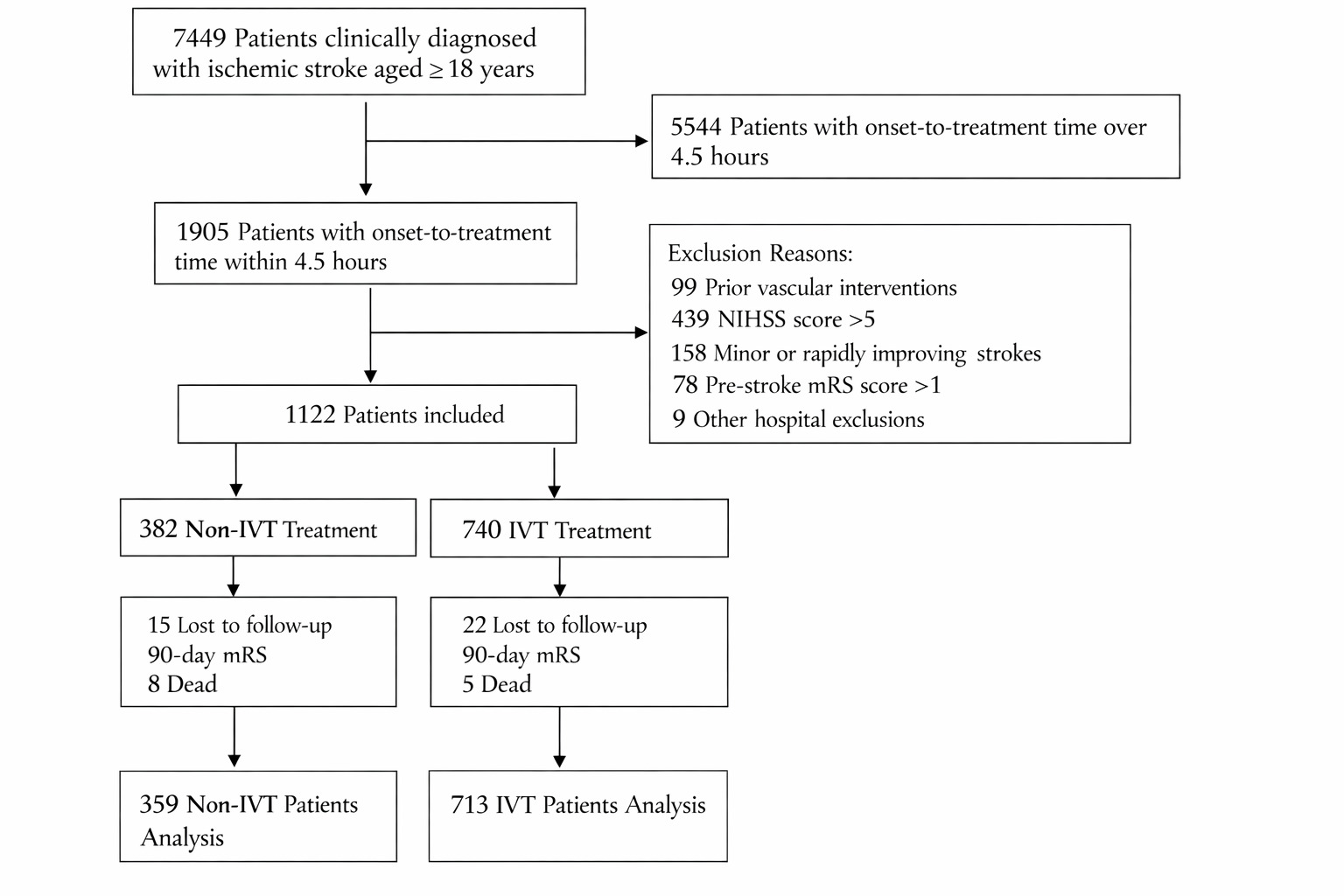

Supplement: Supplementary file 1 [file Image_1.jpg]
